# Topics of Public Interest

**Published:** 1915-03

**Authors:** 


### TOPICS OF PUBLIC INTEREST.

---

**Ethical Problem.** A contributor to the "New Zealand Medical Journal" states that he and another physician were attending different members of a family, apparently without friction, when a third physician was called to attend a third member of the family. The second physician objected on the ground that he himself was the family physician. The editor gives up the problem. In most large American cities, family practice in the old-fashioned sense, has nearly died out. It is pretty generally recognized that the right of choice of the patient or his responsible representative, may be recognized at any time and especially at the beginning of any definite illness. Hence, it is no uncommon occurrence to have different members of the same family under the care of different physicians at the same time and while complaints of infringement of family loyalty, stealing cases, etc., are not infrequent, in a case of this nature, accepting the facts as stated, there would seem to be no prior claim on the part of the second physician, especially since the first did not make one. It is a wise plan to be a good loser.

---

**Regulations As To State Medical Licensure.** A medical diploma is essential except in Massachusetts, Oregon and Tennessee which grant licenses on examination. Otherwise, all states require both diploma and examination (including reciprocity) except New Mexico which recognizes approved medical schools and will license their graduates without examination. All states require at least a four-year high school course except: Canal Zone, Hawaii, Idaho, Massachusetts, Montana, Oregon, South Carolina, Tennessee and Wyoming. The following require, in addition, one year of college work: Arkansas, California, Connecticut, Illinois, Kansas, Kentucky, Michigan, New Hampshire, Oklahoma, Pennsylvania, Utah, Vermont, Virginia, Washington. The following require two years of college work: Alabama, Colorado, Indiana, Iowa, Minnesota, North Dakota, South Dakota. In nearly all states, it is required that the medical course shall consist of four years, although we understand that this is not retro-active. The exceptions in which the length of the college course is not stated are: Canal Zone, Idaho, Massachusetts, Oregon, Philippines, Porto Rico, Tennessee, West Virginia, Wyoming. Many states specify the number of hours per week, total study, length of terms of medical schools, etc. Most states reciprocate with certain other states but as the reciprocity is usually determined by mutual treaty, and as reciprocation has no connection with severity of requirements in general, this matter should be made one of special inquiry by those interested. Except in the Canal Zone, where there is no reciprocity and where a fee of $5 is charged for examination, all states charge at least $10, some as high as $25, for examination. A few make no charge for license by reciprocity, some charge whatever fee the original state has, (Idaho, Illinois and Kansas), most charge the same fee for license by examination or by reciprocity, several charge considerable more—$25, $50, and even, in Utah, $75 for reciprocal license.

**Eugenics Bill for Indiana.** The senate has passed a bill requiring a certificate of health from a physician as a prerequisite for marriage license.

---

**The Harvard Medical School Unit,** will go to Paris to assume charge of 150 beds in the American Ambulance Hospital. Wm. Lindsey of Boston has contributed $10,000 to pay the expenses.

---

**Red Cross—Expirations of Service.** In March, the six-months term of service will have expired and all wishing to do so, will be returned to this country, their place being filled by others.

---

**Belgian Relief Fund**—For Belgian physicians. This is simply a reminder of the sufferings of our confreres. Remittance may be made to American Medicine of N. Y. City directly or through the Buffalo Medical Journal.

---

**Liability of Surgeons for the Negligence of Nurses and Internes:** reversal of the verdict of the lower court in favor of the plaintiff, by the Court of Appeals of Maryland.

Months after a successful operation the family doctor removed pieces of gauze that had been left in the wound, and the professor who had operated was charged with negligence. After reviewing many of the cases, the court agreed with the theory as advanced by the attorneys for the professor, that an operating surgeon at a hospital of repute, in the case of a wound left open is not liable for the negligence of hospital surgeons, nurses or internes, in the after dressing of such wounds, if such operating surgeon is without knowledge of, or not privy to such negligence. The court pictures the effects were this not so, as in many cases a professor would not give his services were he to be held for imperfect dressing of wounds which is usually left in charge of nurses and internes.

Many cases were cited showing negligence at the time of the operation, in allowing foreign substances to remain in the wound but these were distinguished from the case at bar and several old English cases as well as decisions from New Jersey and from the Federal Courts were cited to uphold the conclusion reached by the court.—Frank Engers, 128 S. Bond St., Baltimore, Md.

---

**Railroad Fares.** No reduced rates will be granted on account of the meeting of the State Society in Buffalo. One reason given is the general dissatisfaction with the "certificate plan." With this, no one will dissent. The "certificate plan" is a troublesome device designed to place a purely impersonal, commercial transaction on the basis of a personal courtesy and to prevent the purchaser of a commodity from buying more than he needed for his own use and assuming responsibility for its ultimate sale. Why a straight, round-trip ticket should not be sold, for conventions, with or without precautions against its transfer, does not appear. There has been a general disposition in the last few years to do away with legislation fixing rates of fare, and to raise rates. We think that vertebrae should be prescribed for the members of interstate and public service commissioners and that the general principle should be adopted in a matter so clearly of public utility, of applying a pawl and ratchet device which shall allow rates to be reduced but never increased. Most of the arguments to the contrary overlook the actual experience of the most successful railroads of our own country and of Europe. In particular, comparisons with European experience are usually unfairly expressed in terms of first class fares for the two continents. On the average, first-class American service corresponds pretty closely to third-class English and second- class continental, the service of American railroads being a little better than for third-class English and not quite so good as second-class European—although exceptions occur in either directiion in making particular comparisons. Continental European railways also provide for rates lower than the general average of two cents a mile and we ought also to recognize the needs of a considerable number of persons for cheap transportation, in which the element of transportation itself is far more important than luxury or even comfort.

---

**The Harrison Anti-Narcotic Law** goes into effect March 1. This is a further example of the tendency toward nationalism as compared with the doctrine of states' rights. So far as we can interpret its language, it does not apply to physicians who will content themselves with small therapeutic doses, by single administration or prescriptions of a few doses. Prescriptions containing not over 2 grains of opium, 1/4 grain of morphine, 1/8 grain of heroin or 1 grain of codeine are exempt. Physicians purchasing a stock for themselves or patients must, however, take out a license which includes a pad of special blanks which must be used. The license is obtained from the Commissioner of Internal Revenue at a fee of $1.00 per year, but for the quarter ending the present governmental fiscal year, the fee is 34 cents. It should be understood that anything that approaches dealing in the substances mentioned or that encourages or sustains a drug habit, requires a license, and that any bad faith in the use of these drugs will be severely punished Within a month or two our Medico-Legal Editor, Hon. Henry W. Hill, will publish a more authoritative discussion of the law and of such rulings as may be obtainable.

**The Genesee County Laboratory** at Batavia, under the direction of Dr. F. D. Carr, has been made a depot from which all supplies furnished by the state to health officers and private physicians can be obtained by residents of Genesee and adjacent counties.

---

**The University of Buffalo, Arts Department,** has accepted the generous gift of the building of the Women's Educational and Industrial Union, and will hold classes in that building, on Niagara Square, after March 1. This marks an important step in the achievement of the ambition to establish a college in Buffalo. We take the liberty to point out that fully half of the average college course requires no special equipment. Hence, perhaps by co-operation with the municipal high schools, perhaps by voluntary donation of services of teachers, professional or amateur, complete college courses in modern and ancient languages, philosophic studies such as logic, psychology, etc., pure mathematics and some other branches could be established. The city also has libraries, museums of archaeology and natural history, an art gallery and various semi-public, commercial and private laboratories which, by co-operation, might rival the equipment of the majority of colleges.

---

**Mortality Rate for Registration Area.** Estimating the population from the census of 1910 according to the principles of compound interest, it was 63,298,718 for 1913—about two-thirds of the total for the U. S. 890,848 deaths occurred—14.1 per 1000, the lowest being 13.9 per 1000 for 1912. The lowest rate was 8.5 for Washington, the highest 17.1 for New Hampshire, expleained by the obvious tendency toward westward migration of the young and healthy. The lowest municipal death rate was that of Seattle, 8.4; the highest that of Memphis, 20.8 : 1000. Buffalo, Rochester, Syracuse and Albany had rates of 15.8, 14.6, 15.7, and 19.8, respectively. A generation ago, the standard, normal death rate was considered about 20:1000 and two centuries ago, it was nearly 40:1000, at least for cities. At present, the normal standard is about 15:1000 but as this represents an average longevity of about 60 years—not quite the quotient as the normal population is, so to speak, being constantly diluted by an excess of births over deaths—but it is doubtful whether a population not also diluted by immigration could yet have this low death rate.

Tuberculosis still claims nearly about 10% of the total—1.476 : 1000 population for 1913. Cancer, organic heart disease, nephritis, show a slight increase, as is to be expected as the general mortality decreases and the average longevity increases. The average longevity was 39.8 years, 39.2 for males and 40.6 for females. Nearly 18% of all deaths occur under one year, over 25% under 5 years. A trifle over 1% of all deaths are by suicide, the statistics probably being too low on account of the tendency to cover up this cause of death. Automobiles killed 2,488; other vehicles (mostly horse-drawn but including a few by bicycles and motor cycles) 2,381; animals (mostly horses) 540; railroads 8,212; street cars 1,998. For other vehicles, separately.

---

**Practical Deduction as to Traffic Regulation.** It will be noted that automobiles, in spite of their large numbers and average high rate of speed and the fact that they travel on ordinary high-ways, without guiding tracks, do not kill as many persons as the popular conception holds. They kill less than a third as many as do railroads, only slightly more than street cars, and probably less than horses and horse-drawn vehicles considered together. Yet traffic regulations have, in the last few years been largely framed and mainly inforced against automobiles. The abstract injustice of this is not so important as the result in mortality and injury. We suggest, therefore, a careful analysis, for the future, of accidents connected with traffic, to ascertain not the direct but the ultimate cause. No serious attempt has been made, except in selecting state roads, to consult the needs of modern traffic. In most cities, this is greatly congested, largely because available through routes do not exist, except along streets necessarily occupied by street car tracks, with dangerously narrow space at the sides. In Buffalo, what may be termed trunk streets, affording continuous passage for any considerable distance, for any natural route, occur only at intervals of from 1/2 to 1 mile. There are just about 17 outlets from the city for its 17 miles of land boundary. Few of these are direct and they are more or less combined toward the center of the city. With a few exceptions, for part of the distance, they are congested with street cars and other vehicles. The general principle of this remark applies with equal force to Rochester and many if not most other cities of our territory and to a good many throughout the country. Time, limbs and lives can be saved by establishing adequate trunk streets in cities approximately paralleling street car routes, which have necessarily usurped almost all the trunk routes available.

From a study of near-accidents and of the details of observed and reported accidents, we are confident that careful analysis will show that a large minority, possibly a majority of automobile accidents are due secondarily but essentially to the irresponsibility of pedestrians and horse drivers, fostered by law and its enforcement. Statistics ought to show whether an automobile death was of an occupant of an automobile or of a person struck; whether the chauffeur was drunk or not; whether such contributing or rather essential factors were present as absence of lights on a wagon on a country road, crowding of automobile by a slow-moving vehicle in the middle of the street, driving on left side of street by delivery wagon, garbage wagon, etc., dodging of pedestrian from behind street car into the natural course of other vehicles, child playing tag across street, adult reading or meditating between curbs.

---

**Tonawanda** expects to install a **Chlorin System** for treating the Niagara River water.

---

**The J. N. Adam Memorial Hospital** at Perrysburg, needs a new wing, for which a $60,000 bond issue by Buffalo, is asked. The hospital was planned for 120 adult patients and on Feb. 4, it has 195. Shortly before, the entire waiting list of 30 was admitted and accommodations provided by all sorts of temporary expedients but a new waiting list of 20 speedily formed. A sewage plant is necessary to prevent damage suits from adjoining property holders, an X-ray outfit is almost imperative and kitchens, refrigerators and other equipments must be enlarged. Moving pictures have recently been exhibited, showing children playing naked in the snow—a great contrast to ideas formerly held as to the hygienic treatment of tuberculous patients.

---

**Great Britain and Medical Overcrowding.** Great Britain with a greater ratio of population to physicians, still has the same problem as the U. S. A conservative diminution in the number of students of all grades, in medical courses, has occurred.

---

**Artificial Respiration.** The use of artificial respiration by modern apparatus involving direct inflation, has come to popular attention by reason of the subway accident in New York City and other disasters. We note with pleasure that the Buffalo News has given credit to the originator of this method—Dr. George E. Fell of Buffalo. In view of the controversies that have taken place in the past, it is well to fix this historic point firmly. Dr. Fell wishes us to state that he is not in any way responsible for the recognition of his services by the press.

---

**Reaction of the War on the Journal.** Several of our French exchanges seem to have been discontinued. The copy sent to our editor, Dr. Rene Gaultier of Paris, has been returned marked "Present address unknown to occupants of building." Several copies addressed to physicians in Lille, etc., have come back stamped "Place of destination invaded." And, in view of the general business depression, we take the liberty to ask our readers to impress on advertisers, as much as possible, the value of periodical publicity in legitimate channels, as contrasted with waste-basket advertising.

---

**Public Health Courses.** The Public Health Council has recommended the establishment of short courses in bacteriology, municipal hygiene, water purification, sewage disposal, public health law, etc., in order to secure qualified applicants for positions as health officers. Columbia, Cornell, New York, Syracuse, Buffalo, Union and Rensselaer Polytechnic have been requested to offer instruction.

---

**Reciprocity in Automobile License.** The Adamson bill now before congress and backed by 1½ million automobilists provides that registration in any state shall authorize the use of the car in any other, without further formality.

---

**The Tuberculosis Appropriations of N. Y. State in 1915,** amounted to over 50 millions.

---

**Foot and Mouth Disease.** To the first of January, 110,000 animals were killed to prevent the spread of this disease.

---

**The Sterilization Law** has been pronounced unconstitutional by the U. S. District Court in Iowa.

---

**The Medical Department of Western Reserve** has received a legacy of half a million.

---

**A Bill For a Laboratory For Manufacturing Antitoxin** has been introduced in Missouri, the antitoxin to be distributed free.

---

**The Batavia Hospital** is the recipient of an X-Ray machine kindly presented by Dr. Victor M. Rice of that city.

---

**The U. S. Public Health Service** has issued a constellation chart, showing diagrammatically, the organization of its various activities, which include Domestic (Interstate) quarantine, Marine Hospitals and relief, Sanitary reports and statistics, Foreign and insular quarantine, and Scientific Research, as well as various co-operative activities to which officers are specially detailed.

---

**Disinfection of Excrement on Trains and Vessels.** In 1891, the editor, in view of the threatened invasion of this country with cholera, suggested that all train closets should be fitted with receptacles, to be removed and disinfected at convenient places. Frank, in Public Health Reports, has devised a method by which train and boat closets may be subjected to continuous sterilization by heat of 100 degrees C., by steam or otherwise. This is an important matter which should be put into operation.

---

**Vaccination All The Time.** The Jones-Tallett bill before the N. Y. Legislature would require vaccination only in face of an epidemic. The state health officers are opposing this bill. Small-pox is still an ever-present danger though, like lightning, floods, etc., of course not ever-present at any given place. If vaccination is of value at all—and about 99.40% of those who are in a position to judge, consider that it is—it is needed all the time. If it is not of value, there is no sense in bothering with it when there is an epidemic. We do not believe in the policy of equipping a house with lightning rods after the storm has begun.

---

**The Charity Eye, Ear and Throat Hospital** of Buffalo has presented its annual report for the fiscal year ending September 30, 1914, through Drs. Benjamin H. Grove and George F. Cott. There are 26 surgeons on the active staff, 2981 new patients and 6709 old patients were treated during the year; the total number of treatments being 13,371. 908 operations were performed, 254 on the eye; 654 on the nose, throat and ear. The medical inspection of school children has sent a good many patients to the hospital for relief of ocular defects, removal of adenoids, etc. The law prohibiting imposition on charity is conspicuously displayed on the wall of the waiting room. The profession is invited to refer deserving cases for care.

---

**University Day** was celebrated by the University of Buffalo on Washington's Birthday, as usual, but was of unusual interest. At the Teck Theatre, in the morning, President Charles F. Thwing of Western Reserve, delivered the address, comparing American colleges with European and speaking encouragingly of the prospects of the new Arts Department of the University of Buffalo. The building of the Women's Educational and Industrial Union was formally turned over to the University, for the use of the Arts Dept., the presentation speech being made by Mrs. George W. Townsend, who was chiefly instrumental in the establishment of the Union and who served as president till her departure for the Hawaiian Islands some years ago and who has, ever since, been the Honorary President. Five permanent scholarships were announced for the Arts Department: three for the Women's Union, one by the Women's Investigating Club and one established by Mrs. John Miller Horton in memory of her father, the late Pascal P. Pratt. A dinner was held at the Statler, at which, for the first time, alumni of all departments were represented, 115 being present from the medical department, 275 in all. A constitution was adopted for the Federated Alumni.

